# Understanding the Use and Perceived Impact of a Medical Podcast: Qualitative Study

**DOI:** 10.2196/12901

**Published:** 2019-09-19

**Authors:** Sarah L Malecki, Kieran L Quinn, Nathan Zilbert, Fahad Razak, Shiphra Ginsburg, Amol A Verma, Lindsay Melvin

**Affiliations:** 1 Department of Internal Medicine Toronto General Hospital University of Toronto Toronto, ON Canada; 2 Division of Internal Medicine Department of Medicine Mount Sinai Hospital Toronto, ON Canada; 3 Division of General Surgery Trillium Health Partners University of Toronto Toronto, ON Canada; 4 Division of Internal Medicine Department of Medicine St. Michael's Hospital Toronto, ON Canada; 5 Wilson Centre for Research in Education Toronto General Hospital Toronto, ON Canada; 6 Division of Internal Medicine Department of Medicine Toronto Western Hospital Toronto, ON Canada

**Keywords:** podcasts, grounded theory, medical education

## Abstract

**Background:**

Although podcasts are increasingly being produced for medical education, their use and perceived impact in informal educational settings are understudied.

**Objective:**

This study aimed to explore how and why physicians and medical learners listen to The Rounds Table (TRT), a medical podcast, as well as to determine the podcast’s perceived impact on learning and practice.

**Methods:**

Web-based podcast analytics were used to collect TRT usage statistics. A total of 17 medical TRT listeners were then identified and interviewed through purposive and convenience sampling, using a semistructured guide and a thematic analysis, until theoretical sufficiency was achieved.

**Results:**

The following four themes related to podcast listenership were identified: (1) participants thought that TRT increased efficiency, allowing them to multitask, predominantly using mobile listening platforms; (2) participants listened to the podcast for both education and entertainment, or “edutainment”; (3) participants thought that the podcast helped them keep up to date with medical literature; and (4) participants considered TRT to have an indirect effect on learning and clinical practice by increasing overall knowledge.

**Conclusions:**

Our results highlight how a medical podcast, designed for continuing professional development, is often used informally to promote learning. These findings enhance our understanding of how and why listeners engage with a medical podcast, which may be used to inform the development and evaluation of other podcasts.

## Introduction

### Background

The increasing popularity of medical podcasts in the era of free open access medical education [1-3] has led to a demand for research to evaluate these materials. Evidence based on opinions and consensus from experts [4-6] suggests that educators should consider credibility and podcast length in their listening choices and development of educational materials [4]. A tool for predicting successful anesthesia podcasts has also recently been developed [7] based on literature review because of a paucity of user rating data. There are also expert-defined quality indicators for social media–based research and educational materials [5]. For example, emergency medicine specialists have recently created a system for assessing and curating credible podcasts for graduate medical education [6]. Empirical research on the use of medical podcasts is needed to inform these expert recommendations and future research.

### Podcast User Experience

Despite an increase in podcast development and uptake in medical education, little is known about user motivation and experiences with podcasts as part of ongoing personal learning in medical education and continuing professional development. Particularly, available podcast literature on this topic is largely based on survey data, and it focuses on medical students [8] or residents [9], rather than including practicing medical professionals. The literature that does include practicing physicians is related to social media more generally, including but not limited to podcasts [10-14]. Surveys of medical students or residents have revealed preliminary insights into podcast users’ listening habits, motivation, and perceived impact on practice. For example, a recent survey of medical student listeners suggested that listening while engaging in other activities was common [8]. Another study surveying emergency medicine resident podcast listeners found a primary motivator for listening was to “keep up with current literature” [9]. Despite these insights, survey-based studies limit the depth of responses obtained, and currently, there is limited knowledge on how intrinsic podcast factors, such as content and style, affect user experience.

### Objectives

A greater understanding of how and why individuals across the training spectrum incorporate podcasts into ongoing personal learning in medical education and professional development is required. As an increasingly expanding resource in medical education, a richer understanding of medical podcast consumer experience, from students to independent clinicians, is essential for effective podcast development. Using a locally produced weekly internal medicine podcast as an example of medical podcasting, the objective of this study was to explore how and why physicians and medical trainees listen to podcasts.

## Methods

### Approach

The objective of this study was to identify, through interviews and thematic analysis [15], key factors influencing podcast usage and user experience. The sampling and analytic approach was informed by the principles of constructivist grounded theory, a qualitative methodological approach [16,17]. Web-based podcast analytics were also used to collect preliminary information on podcast use and listening habits, and this information provided the context for qualitative interviews.

### Context

The Rounds Table (TRT) is a free weekly podcast, produced by physicians at the University of Toronto, which summarizes, analyzes, and contextualizes new research in internal medicine. Approximately 100 episodes have been published on the Web since March 2014. Most episodes follow a typical format: 2 cohosts (1 fixed and 1 guest) discuss 2 recently published research studies with broad implications for adult medicine. Each episode concludes with a “good stuff” segment, in which the cohosts briefly recommend something from popular media or scientific literature that has captured their attention and listeners may find interesting. Episodes typically last approximately 30 min. At the time of the study, cohosts were predominantly senior residents or early career staff physicians based at the University of Toronto. The Web-based podcast analytics data were obtained from Blubrry [18], one of the industry’s leading podcast analytics providers. All download, streaming, and play requests were captured to provide a comprehensive statistic of the number of downloads, excluding bots, Web crawler, or machine downloads. This study reported download statistics, including trends over time, geographic distribution of listenership, and listening platforms used.

### Interviews

A total of 21 semistructured interviews with TRT listeners were conducted from June 2016 to March 2017. Purposive and convenience sampling was used to recruit participants varying in geographic location and level of familiarity with the podcast hosts. Listeners were invited first by email in the initial purposive sampling phase and subsequently by announcement during the podcast in the convenience sampling phase. In the purposive sampling phase, 12 of 15 (80%) known podcast listeners invited agreed to participate. In the convenience sampling phase, 10 listeners contacted the podcast hosts to participate after hearing the announcement. Of the 6 medical learners or staff, 5 (83%) were interviewed. A total of 4 volunteers were not medical learners or physicians, and they were therefore excluded after the interview, resulting in a final sample size of 17. All interviews were conducted by the first author (SM), via telephone, Skype audio, or in person, using a semistructured interview guide, which was developed on the basis of group discussion and literature review (Multimedia Appendix 1). The interview guide specifically included questions about TRT podcast, as well as questions regarding general podcast use. Before starting the interview, all participants were asked 4 demographic questions: age, level of training, geographic location of residence, and how many episodes of TRT they had listened to (less than 5, 6-15, more than 15, or all episodes to date). The study was approved by the University of Toronto Research Ethics Board. Participants were offered a nominal gift card for participation.

### Data Analysis

Interviews were recorded and transcribed verbatim. Authors SM and LM, who were not involved with the creation or dissemination of the podcast, analyzed the data using line-by-line coding facilitated by NVivo version 11 (QSR International) to identify initial codes that were subsequently grouped into themes. Analysis proceeded alongside data collection, using an iterative, constant comparative approach [16]. SM and LM met frequently to discuss the evolving coding structure and themes. The interview guide was modified as interviews progressed to elaborate upon themes identified in the earlier interviews. Discussions related to some questions (ie, 1 and 8, Multimedia Appendix 1) were diverse and ultimately did not contribute additional themes or understanding to the data. Data were collected and analyzed until theoretical sufficiency was reached [19]. Subsequently, the coding framework was shared with KQ, NZ, SG, and AAV, who each read a sample of 2 transcripts and provided feedback and comments. The entire team then met to review and finalize the themes. Each member of the research team brought varying perspectives to the analysis. A total of 4 authors (AAV, FR, NZ, and KQ) were the creators or hosts of the podcast, and they may have had preexisting notions about how it was perceived by listeners. To mitigate any potential bias, authors SM and LM, who were not involved in podcast development or dissemination, led the coding and analysis.

### Ethical Approval

The University of Toronto Research Ethics Board reviewed and approved the research (protocol reference #32948).

## Results

### Podcast Use Statistics

**Figure 1:**
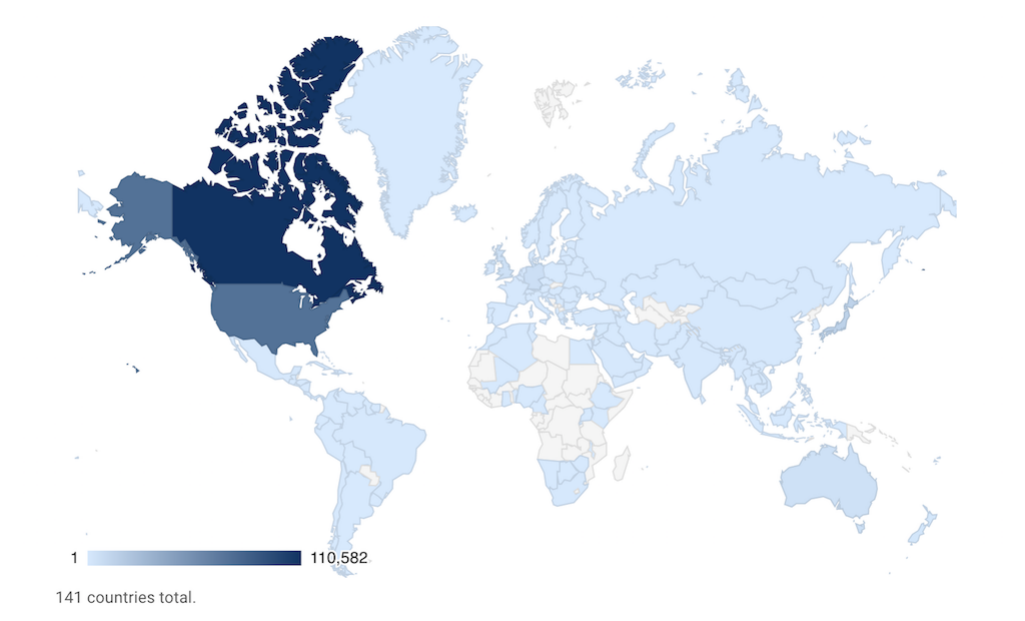
Density map of downloads of The Rounds Table podcast in each country.

TRT has had more than 160,000 unique downloads in 141 countries (Figure 1). More than three-fourths (182,526/227,518, 80.22%) of total downloads are from North America, and the remaining minority are predominantly from Japan (5.92%), Germany (2.69%), the United Kingdom (2.15%), and Australia (2.14%). TRT listenership has grown since its inception, presently averaging 8000 to 10,000 monthly downloads. Most listeners use mobile devices, running Apple iOS or Android operating systems to access TRT (168,363/227,518 total downloads, 73.87%, Table 1).

**Table 1:** Devices used to download The Rounds Table podcast (N=227,518).

Device	Total downloads, n (%)
Mobile	168,060 (73.87)
Desktop	48,253 (21.21)
Other	11,205 (4.92)

### Interview Sample

The sample comprised 8 (47%) men and 9 (53%) women, whose ages ranged from 21 to 49 years. Participants were at varying levels of medical training or practice (2 medical students, 8 residents, and 7 staff physicians). The residents were primarily internal medicine residents, with 1 neurology resident and 1 obstetrics resident. Staff physicians were all internists (general internal medicine or subspecialty trained), with the exception of 1 family medicine–trained hospitalist. Most interviewees (13/17, 76%) resided in Canada, whereas 2 resided in the United States, 2 in the United Kingdom, and 1 in Switzerland. A total of 14 out of 17 (82%) of the interviewees had listened to more than 15 episodes of TRT.

### Themes

Thematic analysis of participant discussions yielded 4 main themes with respect to podcast usage and listenership: to increase efficiency, for keeping up to date, “edutainment,” and to indirectly impact clinical practice. Although our focus was on TRT, participants often discussed podcast use in a more general way, which helped to develop a greater understanding of podcast use in general.

#### Theme 1: Podcast Use to Increase Efficiency

Podcast use was often described as a way of optimizing efficiency, injecting education into otherwise mundane or routine tasks such as commuting, cooking, and cleaning. Many saw this as “multitasking”:

Podcasts are the best way for me to be able to basically study as I go through my morning…

P15, resident

I prefer to listen to [podcasts] during what I would consider to be time which would be otherwise ineffective. So for instance, just commuting to work, or if I have to go for a drive somewhere. And I wouldn’t be doing anything else in that time. I like to be able to put on podcasts and make use of that time and learn something.

P8, resident

Listeners described matching their task to the podcast length. To overcome a mismatch in podcast length to chosen task, 1 participant described listening to TRT at double speed:

They tend to be a good length for what I do. I listen double time so the length tends to be ok for my subway trip.

P4, staff

#### Theme 2: Podcast Use for Keeping Up to Date

Respondents universally described that listening to TRT was a part of their continued efforts to stay up to date in the medical literature:

I find it a very convenient way to continue to keep up with emerging studies and exciting findings in my field.

P5, resident

Some respondents felt that the “journal-club” style format of TRT, in which the podcast hosts critically analyze and discuss the literature, saved them time by eliminating the need to read the article and critique it themselves. Many listeners appreciated the educational value of a critical analysis of recent literature:

I like how they went through each paper in a critical way like you would at a journal club, but it wasn’t presented in such intense detail that you lost the greater picture of what was done or what the results were clinically...In addition to that, the way that some of the papers are presented has helped me be more critical of papers that I read in terms of limitations and strengths.

P10, resident

#### Theme 3: Podcast Listening as “Edutainment”

Participants also reported enjoying the experience of listening because of the entertainment value of TRT, referred to as “edutainment”:

It’s sort of something that I do...it doesn’t feel to me like it’s a lecture. It’s more of a form of entertainment but I’m also at the same time getting educated. So “edutainment.”

P11, medical student

This was reflected in participants’ discussion of their enjoyment in listening to a friendly discussion among colleagues, blended with banter and humor. For example, 1 listener felt a benefit of the conversational style was that it created a sense of “eavesdropping on a conversation” (P2, staff). A conversational style also engendered a sense of familiarity with the hosts. In addition, several participants reported that they either knew one or more of the hosts because of their local reputation or because they were personally acquainted with the hosts. Personal familiarity and local context created a sense of trust or credibility in the information being relayed, as well as a feeling of wanting to support colleagues by listening:

I trusted [Host] that if I listen to this podcast, most likely I am not going to be missing really big landmark trials. So I didn’t have to worry too much not reading Lancet and all these other journals I used to read.

P6, staff

I know a lot of the people that are on it so it seems like if they know this I should know this too-it’s more relevant...the other ones like in the UK, yes it is about geriatric medicine and should be more relevant to my practice but I feel like I don’t really know those people.

P2, staff

As a result of this early finding, the research team actively sought out listeners who did not know the hosts personally to explore the concept of credibility. These listeners reported a varying approach to gauging the credibility of the hosts. Some listeners appeared to determine credibility based on the hosts’ credentials:

I determine their credibility...just from their experience. So, their profiles online. The fact that they’re residents in the field I think just gives them their credibility automatically.

P1, medical student

Others described a comfort with the presentation style and material:

They just sound quite credible, don’t they? [laughing] They sound very comforting...they sound like they know what they’re talking about and they can have a reasonable argument. That to me is more credible than having a list of accolades that you’ve done this x, y and z.

P18, staff

#### Theme 4: Podcast Impact on Individual Practice

Listeners felt that TRT indirectly or over time affected their practice by helping them increase their overall knowledge rather than directly or immediately changing their clinical practice. Specifically, listening to the podcast facilitated their awareness of scientific literature, including major trials and clinical practice guidelines. Listeners described that they may use the podcast as a starting point from which to delve further into a subject or return to read in detail the article discussed on the podcast:

I think it overall just makes you a more well-rounded clinician and not so narrow minded about one way to do things. Makes you come from a different perspective sometimes. So I think that, in a subtle way, likely helps.

P9, resident

Practicing physicians used the podcast to enhance awareness, process scientific information, and determine applicability to clinical practice:

So when I listen to these things does it directly change my practice right away? Probably not because I’m someone who is a little more conservative to my approach in adopting new stuff anyway. But is it information that ultimately changes my mind down the road? For sure...

P4, staff

One physician also noted applicability to teaching:

Sometimes they review papers before they came out [in print]...So I could teach trainees earlier... because I could listen multiple times, I remembered things better so I could apply to my patient care.

P6, staff

On the other hand, trainees described the podcast as a means to develop well roundedness and preparation for potential questions “that you actually get asked about by staff” (P7, resident) on the wards, that is, from supervisors on ward-based rotations:

I remember certain articles that I picked up...being able to recall that and discuss that with my attendings. I don’t know if it actually went to the point where it came down to patient care.

P16, resident

## Discussion

### Principal Findings

This paper explored how and why listeners engaged with a specific medical podcast, TRT, by reporting both Web-based analytics involving more than 160,000 unique downloads and in-depth qualitative interviews with 17 listeners. We found a steady growth in the use of TRT over time, and it can now be classified as a moderate-impact educational intervention [20], suggesting that there is a demand for a general adult medicine podcast. Listeners predominantly engage with the podcast by using mobile devices, which corresponds with our interview findings. We also found that the podcast was perceived to increase efficiency and permit users to multitask by using mobile platforms. This study confirms and extends the findings from survey-based studies of single learner categories [8,9], demonstrating that listeners across all stages of training and practice turn to a medical podcast to expand their knowledge in a way that integrates into their daily lives, enhancing efficiency while simultaneously providing entertainment. We explored intrinsic podcast features that enhance motivation for listening, which has not been previously reported. A key finding of this study was that the enjoyment of listening to a podcast, as well as the consequent motivation for listening, is related to features such as the use of humor, banter, and active critical appraisal of the literature. Listeners of this podcast make credibility judgments based on assumptions related to familiarity and credentials of the cohosts and their presentation style. The results also shed light on what listeners could mean when they say that listening has an impact on their learning and practice [9]. The participants largely used the podcast to augment knowledge, as part of their desire to stay up to date with medical literature. Trainees tended to use the podcast to prepare for ward-based questions from supervisors, whereas practicing physicians used it for teaching or to build a knowledge base and rationale to change future practice. In total, these findings highlight how a podcast developed for continuing professional development is often used informally by its listeners to promote learning.

### Implications for Medical Podcast Development

Although the study focused on 1 podcast in particular, participants also spoke of medical podcast use more generally. Thus, our findings may be useful when considering the development and uptake of medical podcasting in continuing medical education. The finding that podcast use is a way to increase efficiency suggests that medical podcasts should be designed for pairing with common multitasking activities. Similar to other studies [4,21], we found that concise and modular podcasts are preferred. We also found that listeners actively task match, choosing a podcast to suit the length of a task. Our work suggests that in addition to topic complexity [4], discrepancy in listening time preferences may be related to the length of the concurrent task, such as a commute to work, rather than the podcast itself. Podcast developers can use this information to actively design podcast length and features to align with common concurrent tasks to optimize user uptake.

Listeners’ descriptions of using the podcast as a means to keep up to date with the medical literature suggest that podcasts, such as TRT, serve as a supplement in informal medical education. This finding was consistent between medical trainees and practicing physicians. Although podcasts have been used in medical curricula as part of formal education [2], this study reveals that learners across the spectrum of training and into practice also use podcasts as a means to increase overall knowledge, as well as to fill specific gaps that may be identified. Understanding what “gaps” may exist for different populations of medical trainees and clinicians [22] could thus help podcast developers identify content to better target end users. It makes intuitive sense that listeners want to be entertained, especially if they are reaching for a podcast in their downtime. The concept of “edutainment,” the combination of education and entertainment, is popular in educational programming, dating back to television shows such as Sesame Street [23]. The entertainment value of podcasts emerged as a prominent theme in our interviews among medical learners, residents, and staff, extending the findings of an earlier study [9]. Listeners enjoyed active discussions between hosts, along with jokes and banter, which they found to be more engaging than a single host reading a manuscript. Along these lines, they reported enjoying the “good stuff” segment, a portion of the podcast that was included largely for entertainment value. Such intrinsic and modifiable podcast features can be readily adapted across the spectrum of medical podcasts to enhance listener experience and motivation for listening. We did not examine potential differences in the value placed on entertainment between trainees and practicing physicians [12], but this may be an interesting area for further research. The concept of podcast and host credibility was explored in our interviews. Our results suggest that interest and enjoyment in listening itself may engender a sense of familiarity to the hosts, which seems to then lend credibility to the podcast. There may also be intrinsic benefit to having a local podcast, where listeners are more immediately familiar with the hosts from the local context. Interest and familiarity are concepts in current theories on “motivation to learn” [24]. Task value, including interest, is a key influencer of behavior in expectancy-value theory. Self-determination theory outlines intrinsic motivation for a task and the importance of a sense of relatedness or social connection [24]. Further work should explore the interactions among interest, familiarity, and credibility and their relative impact on podcast user motivation to add to existing theory. Creators of medical podcasts may want to consider how to deliberately cultivate a sense of familiarity when a podcast is used beyond a local context. Finally, this study illuminates how the use of a single podcast contributes indirectly to practice by supplementing knowledge. Trainees describe this knowledge in generic terms, whereas practicing physicians describe it as one of multiple sources of potentially practice-changing information. Podcast use is one of several strategies for medical trainees and practicing clinicians to enhance their knowledge. However, the results of this study demonstrate the complexities of using a form of social media for knowledge dissemination. The use of podcasts in informal or multitasking settings may limit their educational impact, as it is not known how engaging in concurrent tasks affects retention of podcast material. In fact, little is known about how podcast information is retained and applied over longer periods [1,8]. Thus, future work should be focused on directly evaluating the effectiveness of continuing professional development podcasts in helping users learn and retain information or skills, such as critical appraisal of scientific literature.

### Strengths and Limitations

Web-based podcast analytics permit comprehensive capture of podcast use statistics; however, they are limited in their ability to understand listener motivation and experience. A strength of this study was using semistructured interviews that elicited rich responses from listeners, allowing us to better contextualize the Web-based data, as well as expand on the mode, purpose, and impact of listening [8,9]. This study’s results are also strengthened by triangulating the experience of multiple listeners, inclusive of trainees and practicing professionals, from different countries. However, the volunteer participants in this research were engaged listeners of a single podcast, which may limit transferability. Future research should explore the experiences with more than 1 medical podcast to determine if the findings of this study can be transferred to another context. The user experiences and motivation of residents versus faculty are also areas for further exploration.

### Conclusions

This study highlights how and why medical trainees and clinicians use a medical podcast in informal medical education. Understanding how emerging technologies can be optimized for medical education and professional development will facilitate design of educational materials at any stage of medical education.

## Appendix

Multimedia Appendix 1 Semistructured interview guide.

## Abbreviations

TRT: The Rounds Table
