# Supplementary material for: Understanding the Use and Perceived Impact of a Medical Podcast: Qualitative Study
Source: JMIR Med Educ. 2019 Sep 19;5(2):e12901. doi: 10.2196/12901 (PMC6754688; doi:10.2196/12901)
Supplement: Multimedia Appendix 1 [file mededu_v5i2e12901_app1.pdf]

Semi-structured interview guide

1. Why did you start listening to The Rounds Table?
2. Why do you continue to listen to The Rounds Table?
3. When do you listen to The Rounds Table?
    a. *On what devices? Do you listen while doing any other activities concurrently?*
    b. *Do you think multitasking impacts your retention of the podcast?*
4. Has listening to The Rounds Table affected your learning? *Tell me more…*
5. Has listening to The Rounds Table impacted your clinical practice? *If yes, please describe how?*
6. Do you listen to any podcasts other than The Rounds Table?
    a. *If yes, which ones?*
    b. *How frequently do you listen to podcasts?*
    c. *How does the Rounds Table compare to other podcasts?*
7. In your experience, how does listening to podcasts differ from reading the literature, such as journal articles?
    a. *When do you reach for a podcast vs. a journal article?*
    b. *How does the format of the podcast (ie, two presenters) affect your listening experience?*
    c. *Does knowing the presenters vs. not knowing them affect your experience or How do they determine whether the presenter is credible, and how does this affect your experience?*
8. Is there anything you don't like about the podcast?
